# The Role of Orientation and Temperature on the Mechanical Properties of a 20 Years Old Wind Turbine Blade GFR Composite

**DOI:** 10.3390/polym13071144

**Published:** 2021-04-02

**Authors:** Mohamed M. Z. Ahmed, Bandar Alzahrani, Nabil Jouini, Mahmoud M. Hessien, Sabbah Ataya

**Affiliations:** 1 Mechanical Engineering Department, College of Engineering at Al Kharj, Prince Sattam Bin Abdulaziz University, Al Kharj 16273, Saudi Arabia; ba.alzahrani@psau.edu.sa (B.A.); n.jouini@psau.edu.sa (N.J.); 2 Department of Metallurgical and Materials Engineering, Faculty of Petroleum and Mining Engineering, Suez University, Suez 43512, Egypt; smataya@imamu.edu.sa; 3 Laboratoire de Mécanique, Matériaux et Procédés (LR99ES05), École Nationale Supérieure D’Ingénieurs de Tunis, Université de Tunis, Tunis 1008, Tunisia; 4 Department of Chemistry, College of Science, Taif University, P.O. Box 11099, Taif 21944, Saudi Arabia; m.hessien@tu.edu.sa; 5 Department of Mechanical Engineering, College of Engineering, Imam Mohammad Ibn Saud Islamic University (IMSIU), Riyadh 11432, Saudi Arabia

**Keywords:** composites, mechanical experiments, lay-ups, potential damage sources, wind turbine materials

## Abstract

This work evaluates the mechanical properties of the glass fiber reinforced polymer (GFRP) material taken from an out of service 100 KW power wind turbine blade which has been in service life of 20 years old. Investigated samples were taken from two positions of undamaged regions at 1.6 m and 5.4 m from the rotor hub, respectively. Microstructure investigation and lay-up analysis were carried out. Fiber weight fraction of the investigated samples was ranging between 0.55–0.60. Tensile and compression tests were carried out at the temperature range from −10 °C to +50 °C on specimens which were machined so as to be loaded in the blade length direction LD, transverse to the blade length TD and off axis; 45° to the blade length. Tensile elastic modulus of the investigated GFRP was determined in the three direction tested. The number of fiber fabric layers found to be decreasing along the blade length away from the root and the density of the fibers along the length is the highest (858 gm/mm^2^) and in the transverse direction is the lowest (83 gm/mm^2^). The microstructure of the GFRP composite showed good wetting for the fiber by the polymer with some features of lack of penetration at the high density fiber bundles and some production porosity in the matrix. The tensile Properties at room temperature (RT) and high temperature are almost similar with the highest properties for the samples aligned with the blade length. The compressive strength is highest at the transverse direction samples and lowest at the blade length direction and decreasing with the increase of the test temperature. The bending properties are significantly affected by the fiber orientation with the highest properties for samples aligned with the blade length and the lowest for the samples with the transverse direction.

## 1. Introduction

The operational conditions and parameters affecting the wind turbine blades require optimization of three material properties that are stiffness, density, and fatigue life [1,2]. The high material stiffness is required to maintain optimal aerodynamic performance, the low density is required to reduce self-loading and gravity forces, and the long-fatigue life is required to reduce material failure and degradation [1]. To meet these requirements, the material of choice is based on fiber-reinforced polymers, wood, and combinations of them [2]. The reinforcements used are mainly fabrics of continuous glass fibers and/or carbon fibers [1,2,3]. The laminated fabrics are combined in layered structure using thermosetting polymers, such as polyester, or epoxy [1,2,3]. This composite material is commonly referred to as glass fiber reinforced polymer (GFRP) [1].

The materials selection [4] of GFRP in this application is based on the requirements of high stiffness, low weight, and long fatigue life [5]. Merit material index for beam M_b_ (M_b_ = E^1/2^ ρ) is depending on the elastic modulus E, and the material density, ρ [1]. The wind turbine blades are subjected to sever service environmental and mechanical loading conditions. Such sever conditions will certainly affect the mechanical properties of the blade material. So that evaluation of the blade material after long life cycle is important for understanding the mechanical behavior and the degradation of this material [6]. Ataya et al. [7] carried out visual inspection on 81 blades of 20 years old wind turbines and defined with allocation of the different forms of discontinuities over the wind turbine blade length. Such damage forms were related to either internal structural defects in the blade materials or outer long life experienced environmental reasons [7,8]. The design life of the modern 5MW wind turbine is proposed to be 20 years [9,10], so that it is important to investigate the wind turbine blade material health at the border of its life. This is of further significance due to the limited investigations of this material and also it can be utilized to reevaluate the proposed service life. In addition, it can be important consideration in the manufacturing of the new wind turbine blades. There are still two particular weaknesses of the laminated fiber-reinforced composites [2], namely; their low tensile and shear strength in the out-of-plane direction and the anisotropic properties as the improvement that promote their stiffness and tensile strength in the fiber direction do not usually have equal beneficial effect on the compressive strength. There are some results for testing of different sizes of the full blade either in new or after-service condition to compare the test results with the design data [11,12,13]. Sayer et al. [14] explored the influence of a wind turbine service life on the mechanical properties of the blade material after service life of 18 years. New blade has been also tested through testing samples from different positions on the blade length. From the conducted tests (tensile, compression, fatigue, and inter-laminar shear strength tests) it has been found that it is not possible to attain clear statement to the aging behavior of the blades, as the results are not statistically relevant. However, it was obvious that degradation occurred in the tension and the compression strength due to service life. However, it is concluded that the material degradation due to static and fatigue testing of the test rotor blade is higher than the material degradation in the service blades due to operation [14]. This conclusion shows the difficulty of exploring the effect of service life on the degradation of the rotor blade.

Recently Zangara et al. [15,16] studied the effect of continuous fibers orientation with respect to the loading direction in composite sandwich core structures under low velocity (4.7 m/s) impact, which is of great consideration under load [15,16]. The effect of heating the matrix is of great importance on the polymer composites, Ferdous et al. [17] found that even self-heating up to 10 °C due to internal friction caused by the increased loading frequency has a considerable effect on the fatigue life of GFRP. In another study on epoxy based concrete, Ferdous et al. [18] found that the compressive loading at temperature higher than 40 °C caused a clear stress drop due to the sensitivity of the epoxy matrix to the temperature.

Wind turbines are designed to operate for a life time of 20 years with normal maintenance and replacing of some parts [10]. In 2016 Beauson and Brøndsted have discussed the end-of-life prospective after the decision of decommissioning of the world first offshore windfarm constructed in Denmark, near Ravnsborg, after turning 25 years [19]. The difficulty is not only located in decommission but also the scenarios after that, which could be one of the following: refurbishment, incineration, or mechanical grinding. Similar end-of-life scenarios are under study for to recycle high amount of tonnage of blades which can reach 2 million tons of by 2050 in USA [20]. Different end-of-life options of the wind turbine blades and their environmental impact have been studied by Liu et al. [21] depend on the blade material and depend also on the type of wind turbine [22].

The difficulties of wind turbine end-of-life processing make it not easy to take such decision. The end-of-life decision should be based mainly on designed life data, running cost compared with the produced power, and close inspection results. There different life prediction using fatigue studies of wind turbine blades which represent the most important structural element of the wind turbine [10]. Other studies are based on fatigue stiffness degradation model [23]. Quasi-static test results can be utilized in prediction the residual stiffness of the blade in subsequent fatigue tests.

From the above discussed literature it can be said that most available works are conducted on the effect of fiber orientation and temperature on the mechanical properties of GFRP were in recently manufactured parts. However, still there is not much data available in the literature reporting the effect of these parameters on the structural and mechanical properties of the blade materials after such long service life. Thus, the aim of this study is to investigate fiber orientation and temperature on the mechanical properties of 20 years old turbine blade material after experiencing this severe service loading and environmental conditions. In this study, the structure of the GFRP will be characterized through identifying the stacking sequence, density of glass fiber layup and investigating the effect of the microstructural discontinuities formed during production of the blade composite material.

## 2. Materials and Methods

### 2.1. Material Sampling

**Figure 1:**
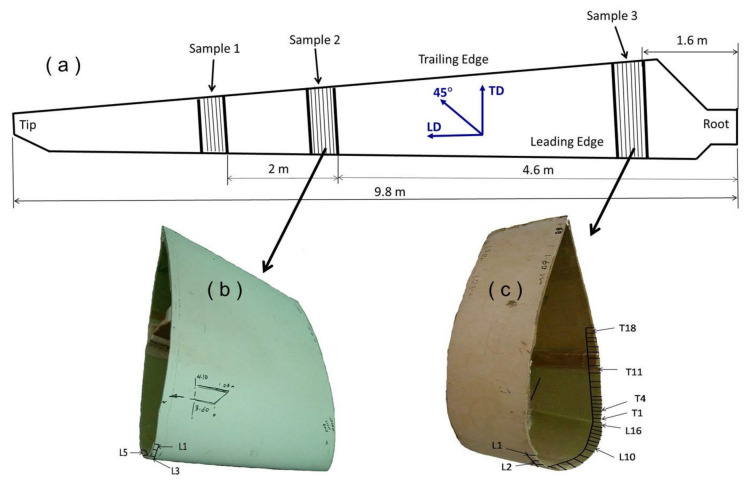
(**a**) Samples location and orientation with respect to the blade length. (**b**) Middle section (Sample 2) of the blade (**c**) Section near to the root (Sample 3).

GFRP samples were cut from a failed blade of a 100 KW wind turbine. The blade is 9.8 m in length and operated for 14 years in the wind turbines site in Hurghada, Egypt, and left out of service for 6 years in the atmospheric conditions. Such working life is nominally equivalent to a number of 6.8 × 10^7^ cycles. In the mentioned site, the average measured wind speed over the year is 6.5 m/s at a height of 24 m. The blade under investigation has an internal longitudinal reinforcing foam web instead of GFRP carrying main spar or webs which are used in most construction of the currently designed blades [24,25,26]. The studied materials were taken from non-broken areas of the blade. Three sample positions were defined (as shown in Figure 1): sample (1) is the thinner test section which is near to the blade tip and at 6.6 m from the root (≈4.8 mm thick), sample (2) is at 4.6 m from the root (≈5.6 mm thick), and sample (3) is taken at 1.6 m from the hub (8–9.2 mm thick).

### 2.2. Characterization Method

#### 2.2.1. Lay-Up Sequence and Microstructure

To investigate the fiberglass fabric layers lay-up, GFRP samples of 100 × 100 mm^2^ were soaked in an electric resistance muffle furnace (Naberthern, Lilienthal, Germany) at 600 °C for 90 min. The samples have been weighted before and after soaking (calcination) to determine the weight fraction of the fibers. The density of the different layers (in gm/m^2^) was determined. Microstructure of the wind turbine blade materials has been investigated on the as-received sample and on the mechanically tested specimens. The samples were cut in a proper size, cold mounted, and prepared according to the preparation procedures for GFRP composites that experienced in Risø DTU (National Laboratory for Sustainable Energy), Technical University of Denmark (TU-Denmark). The samples were ground using SiC papers of 320, 500, 1000, 1200, and 1200 grit size for the times of 1, 2, 4, 6, and 8 min, respectively. Then polished on a 1 μm cloth with the help of 1 μm diamond suspension, this followed by rinsing with water and alcohol cleaned, then air dried. The prepared samples were investigated under Scanning Electron Microscope (SEM) Type Jeol GSM 5410 (Tokyo, Japan) working at 30 KV.

#### 2.2.2. Mechanical Testing

Tension test specimens were prepared according to ASTM D3039 standard in which the specimen dimensions are 25.4 mm (1”) in width and 254 mm (10”) in length. The sample thickness was kept as the sheet thickness of the investigated section. Samples were taken in three directions relative to the blade length, longitudinal direction (LD), transverse direction (TD) and at the diagonal (45°) to the LD. Universal testing machine (Instron-4208–300 kN capacity, Norwood, MA, USA) equipped with a heating furnace up to 700 °C and extensometers was used. Tensile testing was carried out at room temperature (RT), and at 50 °C using a quasi-static strain rate of 0.001 s^−1^. Specimens for three point bending test were prepared according to ASTM D7264 standard. The specimen width was 12.7 mm with a span length between the two supporting points to thickness ratio of 32:1. The studied sample thicknesses (t) were 8 mm and 5.6 mm. The sample length was cut to be 30 t + 50 mm. Samples were taken in three directions relative to the blade length, LD, TD and at 45° to the LD and tested at a cross head speed of 2 mm/s. For each condition, the specimens were tested one time by making the gelcoat to top and other time as gelcoat to bottom. Moreover, compression test samples were prepared according to ASTM D695 and ISO 604 in which the specimen dimensions are 12.7 mm in height and 25.4 mm in width. Samples were taken in three directions relative to the blade length, LD, TD and at 45° to the LD. Compression testing was carried out at different temperatures of −10, 10, 30 and 50 °C at an initial strain rate of 0.001 s^−1^. The temperature was set using an environmental chamber erected on the universal testing machine. The test temperature range was selected (between −10 °C and +50 °C) to include with margin the real temperature range (between 10 °C and +38 °C) of the wind field under investigation.

## 3. Results and Discussion

### 3.1. Lay-Up and Microstructure

**Figure 2:**
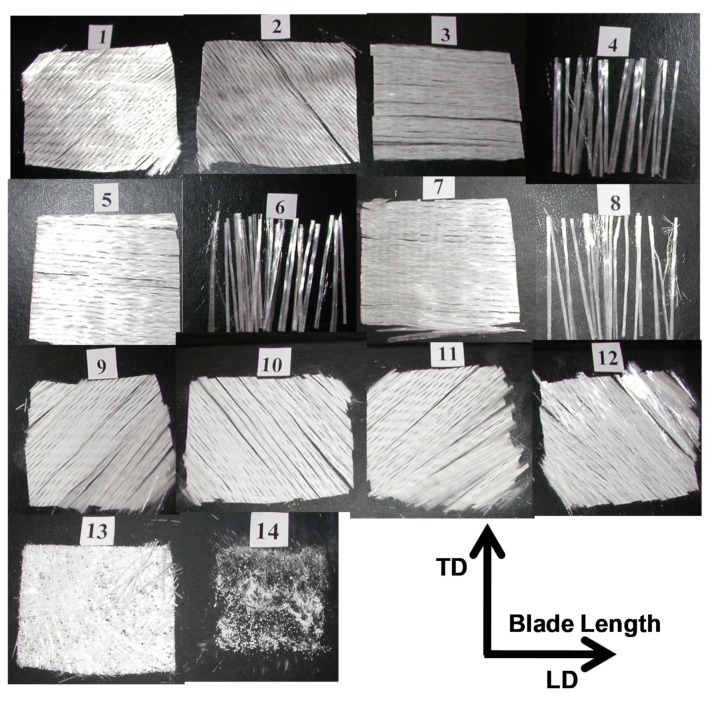
Lay-up sequence of fiberglass fabric layers sequence of S2 (sample 2) (5.6 mm thick) taken at 4.6 m from the root and obtained after sample calcination at 600 °C for 90 min. The numbering from 1 to 13 represent the numbers of glass fiber fabrics layers in the GFRP sample from the internal surface to the outer surface of the blade and 14 is the gelcoat layer.

**Figure 3:**
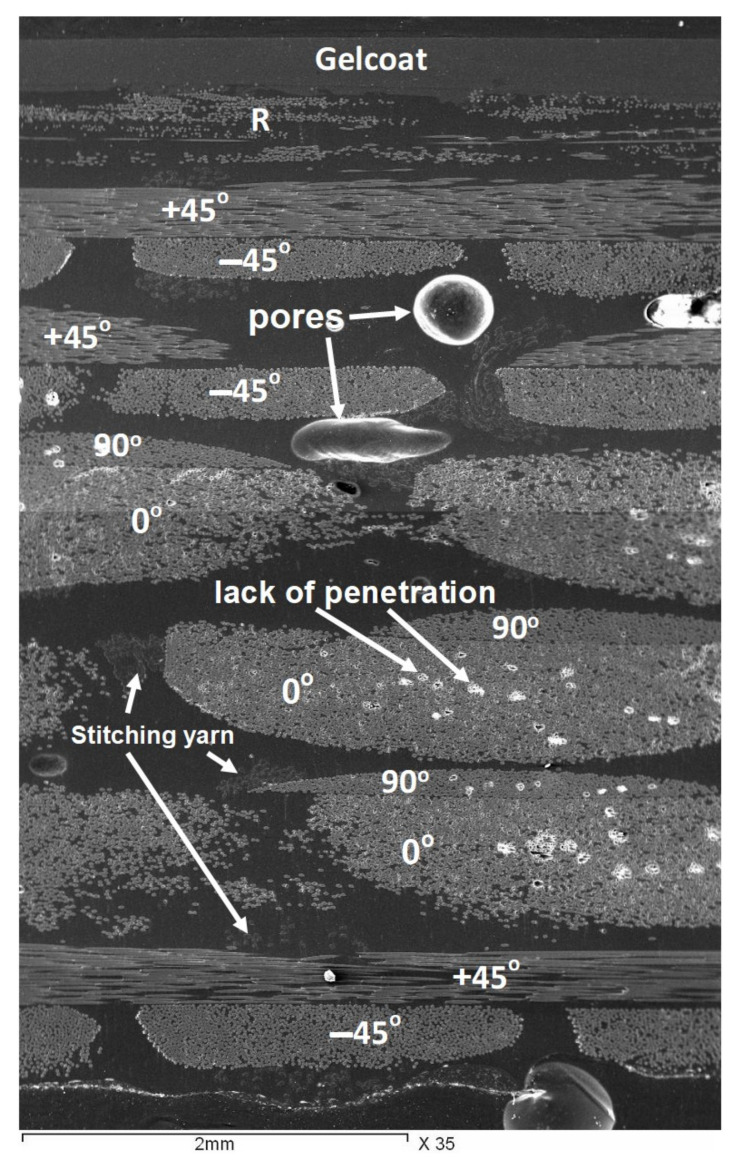
Scanning Electron Microscope (SEM) micrograph for the whole glass fiber reinforced polymer (GFRP) cross section for sample 1.

The mechanical properties of the GFRP are varying depending on many material parameters such as fraction of the reinforcing fibers in the matrix, fiber orientations, density of the used fiber layer fabrics (gm/m^2^) in each orientation, type of matrix and production quality. The wind turbine blade material (GFRP) was close characterized to investigate the effect of such parameters on the mechanical properties. The stacking sequence of the fiberglass fabric layers from the bottom layer which represents the internal surface of the blade to the gelcoating which represents the outer surface is shown in Figure 2, for sample 2. This sample thickness was 5.6 mm, and it was taken from the blade at 4.6 m from the blade root. It can be noted that the total number of fiber fabric layers in this sample is 13 layers with the orientation shown in the figure. The lay-up sequence of the three samples obtained after the calcination process at 600 °C for 90 min is listed in Table 1. It can be observed that the number of fiber fabric layers are varied based on the distance from the blade rotor and it is decreasing as the distance increases. So that the number of the fiber fabric layers found to be 11, 13, and 19 for samples 1, 2 and 3, respectively, which correspond to sample thicknesses of 4.8 mm, 5.6 mm, and ~8.6 mm, respectively. Moreover, the fiber weight percent for the different samples is ranging between 55% and 60%. The GFRP structure from outside surface starts with a gelcoating layer (G) followed by a chopped strand mat of randomly (R) oriented fibers and then the different fiber layer fabrics. The Gelcoat thickness found to be around 0.4 mm. Usually the gelcoat thickness is ranging between 0.25 and 1 mm and in most cases it has a thickness of 0.6 mm [27]. The main functions of the Gelcoat are to protect the blade from ultraviolet degradation, water penetration and to increase the erosion resistance in the sand stormy environments. Filaments of the fiber fabric layer are held together using stitching yarn to keep each filament bundle in its position during the process of manufacturing. Figure 3 shows SEM image for the whole sample cross section starting from the gelcoating layer at the top and the different fiber fabric layers towards the bottom of the image. The dark background represents the polymer matrix, and the features of the different fiber fabric layers are in gray can be observed, as well as the pores, lack of penetration and the stitching yarn. The presence of the stitching yarns in the arrangement shown in Figure 3 indicates that the lay-ups are bidirectional stitched fiber layers. The stitching yarn is burned out during calcination process. The lay-up next to the chopped strand mat is one or more layers of bidirectional ±45° fibers, depending on the GFRP section thickness and location. Then it is followed by a number of bidirectional 0°/90° fibers and ended by one bidirectional ±45° fiber layer. The graded tapering, both in external shape and in thickness of the shells/beams/webs, is usually designed to ensure the same materials loadings, e.g., a maximum strain as design allowable [1]. The change in the density of the various layers can be observed in Figure 2 and from the fibers bundle size shown in Figure 3 which indicates that the fiber density in the blade length LD (0°) is much higher than the density of fiber in the transverse direction TD (90°). The densities of the fibers in the diagonal directions +45°and −45°are equal. A quantitative measurement of the density of the different layers (in gm/m^2^) is presented in Table 2. To increase the rigidity and the load carrying capacity of the blade; which has no carrying spar; the manufacturer tends to increase the number of the 0°/90° which have higher fiber density in the blade length direction LD.

**Figure 4:**
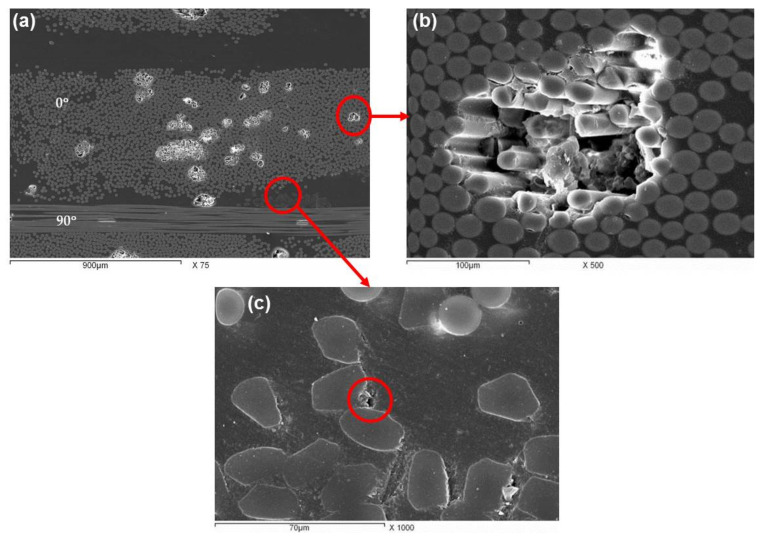
SEM micrographs of as-received wind turbine blade material sample taken in the LD direction. (**a**) Overview (**b**) and (**c**) different magnifications of the glass fibers and stitching yarn, respectively.

Figure 4a–c illustrates higher magnification SEM micrographs with more detailed features for the whole cross section SEM micrograph shown in Figure 3. Figure 4a shows two different orientations of the fiber glass fabric 0° (aligned with the LD of the blade) and 90° (Aligned perpendicular to the LD). It can be observed that the fiber fabric of the 0° orientation has the fiber highest density (858 gm/m^2^ Table 2) to give the blade the required properties (load carrying ability, stiffness, and fatigue resistance), while the 90° orientation has the lowest fiber density (83 gm/m^2^ Table 2) as there are no loads affecting in this direction. One of the whiter features in Figure 4a, observed in the 0° orientation fiber bundle is magnified and presented in Figure 4b. Generally, there is a good wettability for the fibers by the resin, except some little areas inside this high density bundle indicating lack of penetration which appearing as a whiter region. In addition, some larger pores were observed in the matrix regions, Figure 3. Figure 4c shows the circular cross section of the fiber while the stitching yarn which holds the different bundle orientations of the bidirectional oriented fiber bundles (±45° and 0°/90°) have a polygonal shape. Figure 4c includes micro-pores (marked by a circle) around some glass fibers and the stitching yarns too. It is difficult to decide if they lack penetration during production or fibers decohesion due to service fatigue loading of the investigated blade which operated for 14 years (6.8 × 10^7^ cycles). This number of cycles is much higher than the usual fatigue test life of the full blade which assigned to be in the range 2 × 10^6^–2 × 10^6^ cycles according to the guidelines for design of wind turbine [28]. It will be discussed in the next sections if the larger pores and the lack of penetration contribute to the initiation of cracks and failure under tensile loading.

### 3.2. Material Behavior under Tensile Loading

**Figure 5:**
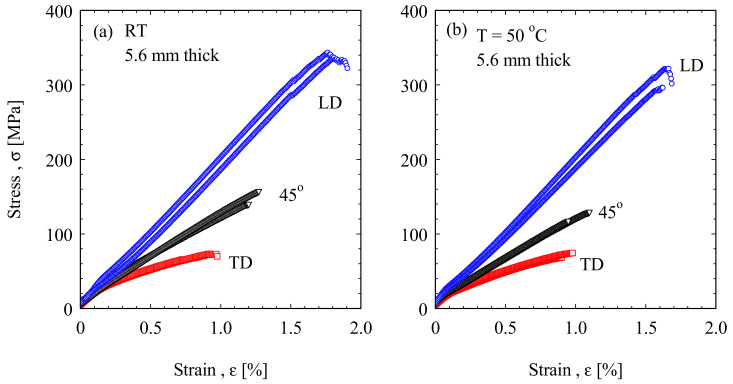
Tensile stress-strain curves of the old wind turbine blade GFRP tested at (**a**) RT and (**b**) 50 °C.

**Figure 6:**
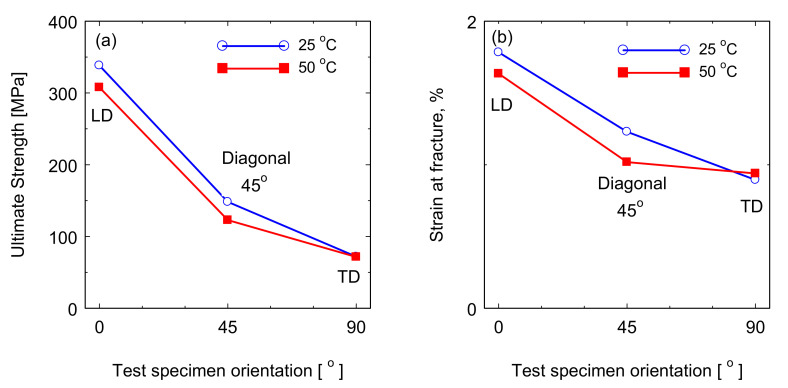
The relation between the tensile properties of the wind turbine blade composite materials tested at room temperature (RT) and 50 °C and the sample orientation relative to the blade length (**a**) ultimate tensile strength (**b**) strain at fracture.

Figure 5 shows the stress-strain curves for the blade material loaded at the three different directions LD, TD, and 45° at room temperature and 50 °C. A clear anisotropic property behavior of this material is shown from the different stress levels. It can be observed that the samples taken parallel to the blade length LD (0°) have the highest tensile strength at both temperatures. This is mainly due to the alignment of the reinforcing fiber with the tensile force axis. However, the samples taken perpendicular to the LD have the lowest tensile strength. This is mainly due to the alignment of the reinforcing fiber perpendicular to the tensile force axis. As it was expected, the results of the samples taken at 45° have tensile strength at intermediate values relative to the other two directions (LD and TD). This anisotropic behavior of fiber reinforced plastics is consistent with the density and direction of fiber within the blade materials. The ultimate tensile strength and tensile strain at fracture for the curves in Figure 5 for the different sample orientations at RT and at 50 °C are plotted in Figure 6.

The elastic modulus was calculated and found to be about 15.6 GP for the specimens loaded in the longitudinal direction. Whereas the elastic modulus for the specimens loaded in TD and 45° is found to be 7.4 and 10.9 GPa, respectively. These values are much lower than the reported values of the new GFRP (26.7 GPa in LD and 9.2 GPa in the TD) [29]. However the long working life of the investigated materials (14 years, 6.8 × 10^7^ cycles) can result in stiffness degradation and reduction of the elastic modulus [5,30]. In testing of GFRP containing 36 wt% fibers the elastic modulus was decreased from 24.6 GPa to ~19 GPa after fatigue loading life of 4 × 10^5^ at R = 0.1 [29,30]. The attained tensile strength in this study at room temperature was around 350 MPa in the longitudinal direction is lower than the values (achieved for samples from 100 KW blades [14], whereas the fracture strain is comparable with the values in the same reference. This results confirms the statement that the glass fibers reinforced composites have a moderate tensile properties among other reinforcing materials such as natural or carbon fibers [31].

**Figure 7:**
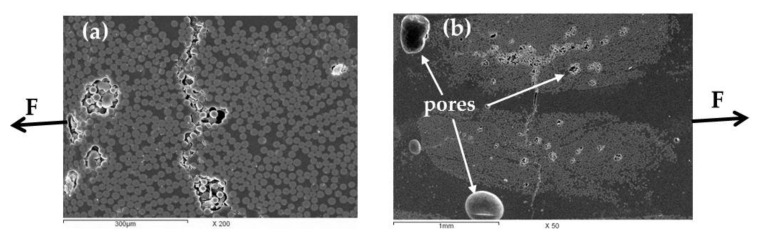
SEM micrograph for some fracture features observed in the fractured tension test sample of wind turbine blade material showing: (**a**) crack through fibers’ bundle and (**b**) manufacturing pores are not the origin of the cracks.

The damage features of the tensile samples after testing is shown in the SEM micrographs in Figure 7. Under tensile load, the cracks were started from the lack of penetration areas and grown perpendicular to the tensile loading direction (F). A coalescence of initiated cracks was taken place in the fiber bundles and passed through the matrix to other fiber bundles (Figure 7b). Although the large matrix pores (which can be seen as a production defect) are located near to the crack pass or the highly loaded area, no cracks were observed to originate from these pores. It means that the pores cannot be described as a primary cause for the failure. In addition, fracture of the glass fibers aligned with the loading direction was also frequently observed.

### 3.3. Compression Test Results

**Figure 8:**
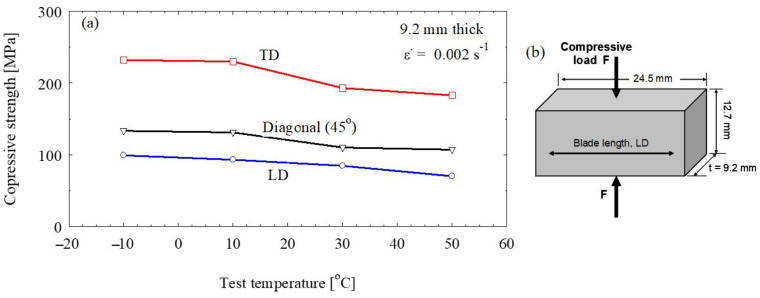
(**a**) Compressive stress–strain flow curves of wind turbine blade materials tested at various temperatures. (**b**) Schematic drawing for the compressive load position relative to the blade length upon testing.

Polymers are usually sensitive to temperature [32,33], so that is important to explore the temperature effect on the mechanical properties. Compression loading of GFRP at various temperatures (−10–50 °C) showed that the samples taken in the TD direction (90°) have the highest compressive strength at all temperature (Figure 8). This is because most reinforcing fibers are aligned parallel to the applied force. However, the samples taken with the blade length (LD or 0°) has the lowest compressive strength at all temperatures, where most fibers are extending perpendicular to the applied force. At the tested temperature range, slight temperature effect was observed in LD and 45° samples. Generally, the compressive strength of the GFRP is higher than that of other competitive reinforcing materials such as natural or carbon fibers [31].

### 3.4. Three-Point Bending Behavior

**Figure 9:**
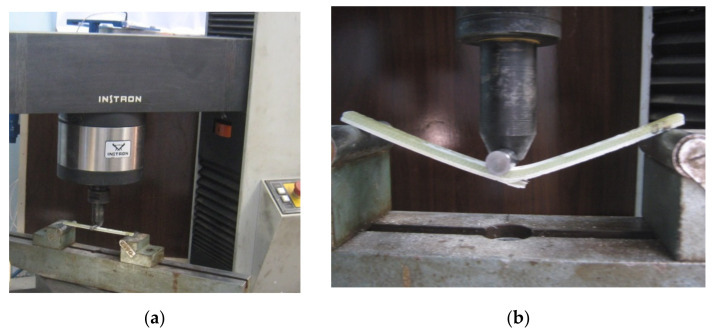
Three-point bending test of glass fiber reinforced polymer (GFRP) sample (S2) 5.6 mm (**a**) at the test start, and (**b**) at fracture.

**Figure 10:**
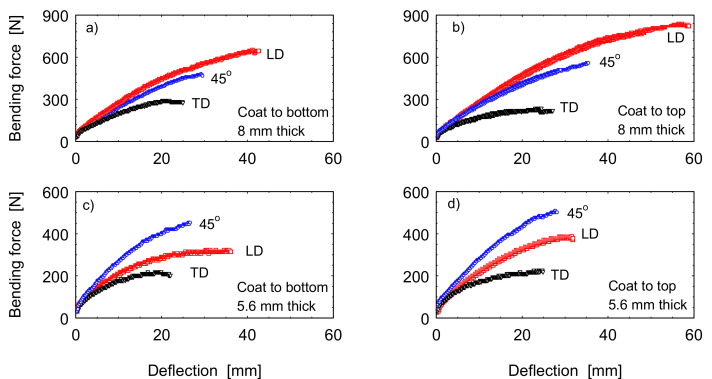
Effect of sample orientation and the gelcoating on bending load—deflection curves of the blade materials (**a**,**b**) samples S3 (8 mm thick) and (**c**,**d**) S2 (5.6 mm thick).

**Figure 11:**
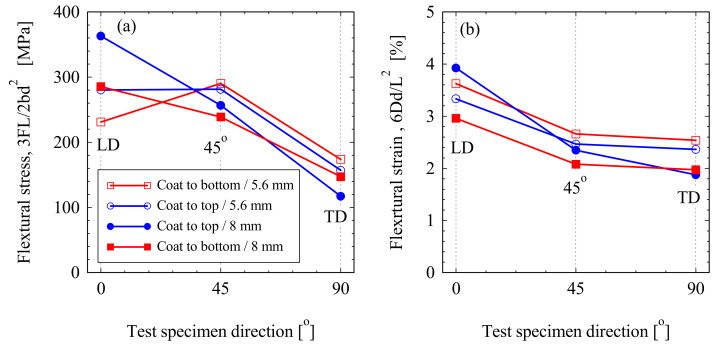
Effect of sample orientation and gelcoating of the blade materials on (**a**) bending maximum stress and (**b**) bending strain at maximum deflection. Note: Legend is the same for (**a**) and (**b**).

Three-Point bending test assembly of the GFRP at the start of the test and after the sample loaded till fracture is shown in Figure 9a,b respectively. The test has been conducted for GFRP material from sample 2 and 3. Figure 10 shows the flexural bending load represented against the deflection of the GFRP tested samples. It can be noticed that, for the same sample, higher bending load was reached when the gelcoat was towards the top. Although the span distance *L* was changed in relation to the specimen thickness (*L* = 32 t mm) it has shown that higher deflection was achieved in the thick sections. The maximum flexural stress *σ_f_* and flexural strain *ε_f_* are calculated according to the formula indicated in ASTM D790 and ISO 178 for rectangular section:

(1) $$\sigma_{f}=\frac{3PL}{2bd^{2}},\;\;\;\varepsilon_{f}=\frac{6Dd}{L^{2}}$$

where *P* is the load, *L* is the support span, *b* is the width of test section, *d* is the height of test section, and *D* is the maximum deflection at the beam center. The maximum flexural bending stress and strain are presented in Figure 11. Logical arrangement of the bending load with the sample orientation can be shown in the thick section, Figure 10. Where higher load carrying capacity was observed in specimen taken from the thicker sample, which located near to the blade root. Six layers of the bidirectional stitched fabric 0°/90° of the highest density fibers in the LD (0°) direction and only two layers from the bidirectional stitched fabric ±45° of the equal density in both directions leads to increased load carrying capacity of the composites in the blade length. Whereas, away from the blade root, the number of the thinner reinforcing layer (0°/90°) in the blade length LD decreases to reach only three layers against three layers of the equal density bidirectional stitched fabric ±45° in the S2 (sample 2) of the thickness of 5.6 mm. The flexural strain in percent for the samples tested in the different orientations is presented in Figure 11b. specimens oriented in the blade length (LD) have shown the highest strain in the different samples, while the TD specimens have indicated the lowest strain. Higher strain was attained on loading thinner specimens (S2) than on loading the specimens (S3) which taken at 1.6 m from to the root.

**Figure 12:**
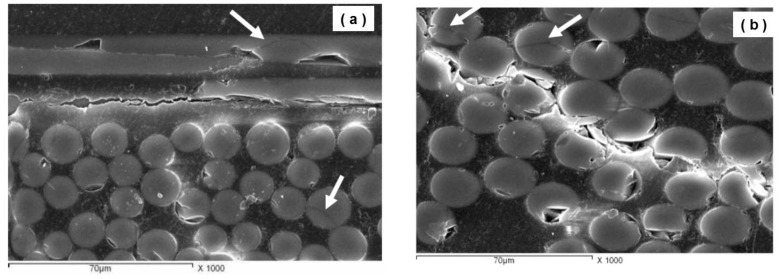
SEM micrograph of fractured bending test sample (S3, 8 mm thick) of wind turbine blade material showing fibers fracture; (**a**) through matrix cracking, and (**b**) through fibers bundle cracking.

Figure 12 shows the SEM micrograph of the bending test sample (S3, 8 mm thick), it shows cracking through the matrix area between the fiber layers (Figure 12a). Moreover, the fibers extending parallel to the specimens length showed partial fiber fracture (as indicated by the arrows), these lead finally to delamination of the GFRP section. Shear at 45° to the loading direction through the glass fiber bundles extending transverse to the specimen length with cracking at around 45° to the loading direction (Figure 12b). In addition, fiber fracture and fibers cracking (indicated by the arrows) were observed around the path of the main crack.

### 3.5. Effect of Fibers Orientation

The advantage of construction with GFRP is to benefit from the ability to increase load carrying capacity in the fiber direction which is known as the tailorability available in composite materials in general. Due to this strong tailorability, composite materials can be designed to satisfy the needs of technologies related to the energy, construction, aerospace, automobile, and other industries. Thus, composite materials constitute most of the commercial engineering materials nowadays [34,35,36]. On the other hand, this can result in anisotropy of the evaluated mechanical properties due to the different concentration of fibers in the different directions. As shown in Table 1 the internal lay-up are mainly with the arrangement of 0°/90° with the maximum density for the 0° lay-up which reaches 858 gm/m^2^, while the 90° lay-up is of low density (83 gm/m^2^). This results in the highest strength being in all loading conditions of this GFRP maximum on the longitudinal direction of these composites. Gelcoat fractures rapidly when found on the lower fiber of the bending specimens and this accelerates the total fracture of the GFRP. In terms of the tensile loading the samples aligned with the LD of the blade gave the highest tensile properties and this is mainly due to the high density of the fiber oriented in this direction that enhances the load carrying capacity in this direction. The samples aligned with the TD gave the lowest tensile properties due to the low density of fibers oriented with this direction. As expected the samples at 45° at average level between the two cases. This behavior is mainly because in the tensile loading the fibers are the main carrier for this load, and thus the tensile properties directly affected by the fiber orientation and density. This behavior is slightly different in case of the compressive test results as in this case, the matrix has a role with the fiber and once the matrix failed the sample will fail in total. Thus in compression, the samples aligned with the TD direction gave the highest compressive strength and that aligned with the LD gave the lowest compressive strength. In the bending test results another factor play a role which the gelcoat towards the top or towards the bottom. It can be noted that when the gelcoat towards the bottom this gave low bending strength as the gelcoat is brittle and fracture quickly and play as a crack initiation for the GFRP that accelerate the fracture and reduce the bending forces.

### 3.6. Effect of Temperature on the Composites Mechanical Properties

In case of the GFRP, the temperature mainly affects the matrix material and make it softer, while the fiber as a thermally stable material expected not to be affect by the range of temperatures examined Although the selected range of test temperature (up to 50 °C) is still away from the softening temperature of the epoxy, the matrix it seems affected by increasing the testing temperature specially in case of the compressive loading. The compressive strength for the samples in all directions is reduced slightly by increasing the test temperature (Figure 8). Thus, it is expected the prolonged exposure for this temperature can affect the compressive loading behavior. On the other hand, the tensile testing result showed almost no effect for this range of temperatures on the tensile properties in the samples examined in all direction. Of course, the tensile loading is more important to be safe in this instance due its effect on the crack propagation. Ferdous et at. [18] examined epoxy matrix concrete at different temperatures and found that the clear softening takes place at 60 °C and higher.

The investigated composite material was taken from a blade of a failed turbine due control defect not due to blade failure, while other 25 turbines of similar blades are still operating in Hurghada wind field. The failure mode of the tested samples was mainly related to the test loading not due to any manufacturing defects. Inspection results [7,8] of the wind turbine blades have showed some cracks at some critical geometrical change region. A follow up inspection after one year did not show development of such stable cracks. The operation team is facing the normal running and maintenance in repair and replacement of some mechanical part.

Decommissioning of the wind turbines could be decided based on environmental, safety, and economic studies of wind field. Moreover, there are some opinions in the Egyptian Authority for Renewable Energy to keep this wind site containing 100 kW and 300 kW turbines as a memorial site. So that, periodical inspection is recommended to detect any serious damage originated from the early found discontinuities or due to any new issue.

## 4. Conclusions

In the current study the fiber fabric lay-up, microstructure, tensile properties, compressive proprieties, bending properties of A 20 years old wind turbine blade GFRP material have been investigated and the following conclusions can be drawn:

(1) The number of fiber fabric layers is decreasing along the blade length away from the root and the density of the fibers along the length is the highest (858 gm/mm^2^) and in the transverse direction is the lowest (83 gm/mm^2^).
(2) The microstructure of the GFRP composite showed good wetting for the fiber by the polymer with some features of lack of penetration at the high density fiber bundles and some production porosity in the matrix.
(3) The tensile properties at RT and high temperature are almost similar with the highest properties for the samples aligned with the blade length.
(4) The compressive strength is highest at the transverse direction samples and lowest at the blade length direction and decreasing with the increase of the test temperature.
(5) The bending properties are significantly affected by the fiber orientation with the highest properties for samples aligned with the blade length and the lowest for the samples with the transverse direction. The bending properties also affected by the gelcoat layer and the thickness of the sample.
(6) Flexural loading has led to the following damage features: fiber fracture, through matrix cracking and cracking the fibers bundle.

Future work: The investigated wind field still has 25 working wind turbines (100 kW) with blades similar to the current investigated one. So that, further studies will be carried out to determine the degradation date of the wind turbine blades and to indicate the feasibility of leaving those working wind turbine operating based on the findings of the current studies and the severity of detected discontinuities in the previous studies.

## Figures and Tables

**Table 1:** Fiber fabric lay-up sequence in the GFRP from internal to external surface *.

Samples	Fiber Density (wt.%)	Lay-Up Sequence of GFRP										
		1,2	3,4	5,6	7,8	9,10	11,12	13,14	15,16	17,18	19,20	
Sample 1 (S1)	59	±45°	0°/90°	0°/90°	0°/90°	±45°	R/G	−	−	−	−	
Sample 2 (S2)	60	±45°	0°/90°	0°/90°	0°/90°	±45°	±45°	R/G	−	−	−	
Sample 3 (S3)	55	±45°	0°/90°	0°/90°	0°/90°	0°/90°	0°/90°	0°/90°	±45°	±45°	R/G	

* ±45°: Equal density bidirectional stitched fibers, 0°/90°: Bidirectional stitched fibers, much denser in 0° (LD) than in 90° (TD) direction, R: Chop strand mat of random oriented fibers, and G: Gelcoat.

**Table 2:** Fiber density of the different layers and the individual orientations from GFRP calcination.

Orientation	Random	+45°	−45°	0°	90°
Fibers density (gm/m^2^)	161	598	598	858	83

## Data Availability

The data presented in this study are available on request from the corresponding author.

## References

[1] Brøndsted P., Lilholt H., Lystrup A. (2005). Composite materials for wind power turbine blades. Annu. Rev. Mater. Res..

[2] Hayman B., Wedel-Heinen J., Brøndsted P. (2008). Materials Challenges and Future Wind Energy. MRS Bull..

[3] Mishnaevsky L., Branner K., Petersen H.N., Beauson J., Mcgugan M., Sørensen B.F. (2017). Materials for Wind Turbine Blades: An Overview. Mater. Rev..

[4] Ashby M.F. (2011). Materials Selection in Mechanical Design.

[5] Sutherland H. (1999). Properties of Wind Turbine. Wind Energy.

[6] Akay B., Ragni D., Simão Ferreira C.J., van Bussel G.J.W. (2013). Experimental investigation of the root flow in a horizontal axis wind turbine. Wind Energy.

[7] Ataya S., Ahmed M.M.Z. (2013). Damages of wind turbine blade trailing edge: Forms, location, and root causes. Eng. Fail. Anal..

[8] Ataya S., Ahmed M., Ahmed E. (2018). An Investigation of Damages in Low Power Wind Turbine Blades. J. Pet. Min. Eng..

[9] Meng H., Lien F.S., Glinka G., Geiger P. (2019). Study on fatigue life of bend-twist coupling wind turbine blade based on anisotropic beam model and stress-based fatigue analysis method. Compos. Struct..

[10] Nijssen R.P.L., Brøndsted P., Brøndsted P., Nijssen R.P.L. (2013). Fatigue as a design driver for composite wind turbine blades. Advances in Wind Turbine Blade Design and Materials.

[11] Kensche C.W. (1996). Fatigue of Materials and Components for Wind Turbine Rotor Blades.

[12] Bürkner F., Van Wingerde A. Testing of rotor blades. Proceedings of the 8th International Conference on Structural Dynamics (EURODYN).

[13] Bintu A., Vincze G., Picu R.C., Lopes A.B. (2016). Effect of symmetric and asymmetric rolling on the mechanical properties of AA5182. Mater. Des..

[14] Sayer F., Bürkner F., Buchholz B., Strobel M., Wingerde A.M., Busmann H., Seifert H. (2013). Influence of a wind turbine service life on the mechanical properties of the material and the blade. Wind Energy.

[15] Zangana S., Epaarachchi J., Ferdous W., Leng J. (2020). A novel hybridised composite sandwich core with Glass, Kevlar and Zylon fibres—Investigation under low-velocity impact. Int. J. Impact Eng..

[16] Zangana S., Epaarachchi J., Ferdous W., Leng J., Schubel P. (2021). Behaviour of continuous fibre composite sandwich core under low-velocity impact. Thin-Walled Struct..

[17] Ferdous W., Manalo A., Peauril J., Salih C., Raghava Reddy K., Yu P., Schubel P., Heyer T. (2020). Testing and modelling the fatigue behaviour of GFRP composites—Effect of stress level, stress concentration and frequency. Eng. Sci. Technol. Int. J..

[18] Ferdous W., Manalo A., Wong H.S., Abousnina R., AlAjarmeh O.S., Zhuge Y., Schubel P. (2020). Optimal design for epoxy polymer concrete based on mechanical properties and durability aspects. Constr. Build. Mater..

[19] Beauson J., Brøndsted P., Ostachowicz W., McGugan M., Schröder-Hinrichs J.-U., Luczak M. (2016). Wind Turbine Blades: An End of Life Perspective. MARE-WINT: New Materials and Reliability in Offshore Wind Turbine Technology.

[20] Cooperman A., Eberle A., Lantz E. (2021). Wind turbine blade material in the United States: Quantities, costs, and end-of-life options. Resour. Conserv. Recycl..

[21] Liu P., Meng F., Barlow C.Y. (2019). Wind turbine blade end-of-life options: An eco-audit comparison. J. Clean. Prod..

[22] Kouloumpis V., Sobolewski R.A., Yan X. (2020). Performance and life cycle assessment of a small scale vertical axis wind turbine. J. Clean. Prod..

[23] Liu H., Zhang Z., Jia H., Li Q., Liu Y., Leng J. (2020). A novel method to predict the stiffness evolution of in-service wind turbine blades based on deep learning models. Compos. Struct..

[24] Habali S.M., Saleh I.A. (2000). Local design, testing and manufacturing of small mixed airfoil wind turbine blades of glass fiber reinforced plastics: Part I: Design of the blade and root. Energy Convers. Manag..

[25] Manwell J.F., McCowan J.G., Rogers A.L. (2006). Wind Energy Explained: Theory, design and application. Wind Eng..

[26] Subrahmanian K.P., Dubouloz F. (2009). Adhesives for bonding wind turbine blades. Reinf. Plast..

[27] Yuhazri M.Y., Sihombing H., Zaimi Z.A.M., Nilson G.C.H. (2015). A review on gelcoat used in laminated composite structure. Int. J. Res. Eng. Technol..

[28] (2013). Guidelines for Design of Wind Turbines.

[29] Saribiyik M., Caglar N. (2003). Determination of mechanical properties of pultruded grp box section using a “short” tensile coupon. Gazi Univ. J. Sci..

[30] Salekeen S., Jones D.L. (2007). Fatigue response of thick section fiberglass/epoxy composites. Compos. Struct..

[31] Javaid U., Khan Z.M., Khan M.B., Bassyouni M., Abdel-Hamid S.M.S., Abdel-Aziz M.H., Ul Hasan S.W. (2016). Fabrication and thermo-mechanical characterization of glass fiber/vinyl ester wind turbine rotor blade. Compos. Part B Eng..

[32] Abdel-Wahab A.A., Ataya S., Silberschmidt V.V. (2017). Temperature-dependent mechanical behaviour of PMMA: Experimental analysis and modelling. Polym. Test..

[33] Guo H., Lu C., Chen Y., Tao J., Chen L. (2018). Thermal—Mechanical Coupling Behavior of Directional Polymethylmethacrylate under Tension and Compression. Polymers.

[34] Chung D.D.L. (2010). Composite Materials Science and Applications.

[35] Zayed E.M., El-Tayeb N.S.M., Ahmed M.M.Z., Rashad R.M. (2019). Development and Characterization of AA5083 Reinforced with SiC and Al2O3 Particles by Friction Stir Processing. Advanced Structured Materials.

[36] Bakkar A., Ahmed M.M.Z., Alsaleh N.A., Seleman M.M.E., Ataya S. (2019). Microstructure, Wear, and Corrosion Characterization of High TiC Content Inconel 625 Matrix Composites. J. Mater. Res. Technol..

